# Editorial: Disparities in early onset colorectal cancer

**DOI:** 10.3389/fonc.2024.1537473

**Published:** 2024-12-19

**Authors:** Caitlin C. Murphy, Swati G. Patel, Peter S. Liang, Jennifer M. Weiss

**Affiliations:** ^1^ School of Public Health, University of Texas Health Science Center at Houston, Dallas, TX, United States; ^2^ Department of Medicine, Division of Gastroenterology & Hepatology, University of Colorado Anschutz Medical Center, Aurora, CO, United States; ^3^ Department of Medicine, Rocky Mountain Regional Veterans Affairs Medical Center, Aurora, CO, United States; ^4^ Department of Medicine, NYU Grossman School of Medicine, New York, NY, United States; ^5^ Department of Population Health, NYU Grossman School of Medicine, New York, NY, United States; ^6^ Department of Medicine, VA New York Harbor Health Care System, New York, NY, United States; ^7^ Division of Gastroenterology and Hepatology, University of Wisconsin School of Medicine and Public Health, Madison, WI, United States

**Keywords:** early onset colorectal cancer, disparities, cancer risk factors, cancer genetics, cancer

Early onset colorectal cancer (EOCRC)—defined as colorectal cancer diagnosed before 50 years of age—is on the rise, with an alarming, worldwide increase in incidence rates over the past two decades, particularly in developed countries (1). In the United States, substantial disparities exist in EOCRC. Over the past 30 years on average, Non-Hispanic American Indians/Alaska Natives have seen the highest incidence rate per year of EOCRC compared to Non-Whites (15.2 versus 10.5 per 100,000) (2) while Non-Hispanic Blacks have the highest mortality rate per year (4.2 versus 2.7 per 100,000) (3). A recent Delphi initiative statement on international management guidelines for EOCRC (DIRECt) was published and endorsed by four scientific societies (4). The guidelines cover evidence-based statements on diagnosis, risk factors, genetics, pathology, treatment, and supportive care for individuals with and survivors of EOCRC. The Research Topic, “*Disparities in Early Onset Colorectal Cancer,”* digs deeper and explores existing disparities across the continuum of prevention, diagnosis, treatment, and survival of EOCRC with a global perspective, in hopes of offering ways to mitigate the inequities.

Dwyer et al. summarize an international think tank convened to address the global phenomenon of EOCRC and propose strategies to move critical research forward on ways to reduce incidence, mortality, and disparities in EOCRC. Four key areas of research and intervention focus were identified during the think tank: (1) population adherence to screening guidelines, (2) utilization of emerging screening modalities, (3) understanding family history of CRC, and (4) determining the etiologic mechanisms of EOCRC. In addition, within each of these areas, health disparity considerations for further research are outlined. The information from this paper will help to prioritize the research agenda on reducing disparities in EOCRC.

Two important concepts in the prevention of EOCRC include understanding the role of exposures in the etiology of EOCRC and screening programs to help prevent EOCRC or identify early cancers. Wang et al. explore temporal trends in EOCRC and early life exposures at the country-level using data from the *Global Burden of Disease, Injuries, and Risk Factors 2019*. They focus on the hypothesis that early life is an important window of susceptibility for developing EOCRC and analyze aggregate associations of exposures in previous decades or during childhood and adolescence. Similar to prior studies, they observed higher incidence rates in countries or regions with higher socioeconomic status based on the social demographic index (SDI) and gross domestic product (GDP) per capita, as well as a substantial upward trend in countries or regions with rapid socioeconomic development. They also noted significant country-level associations of GDP per capita, SDI, and summary exposure values of iron deficiency, high body mass index, suboptimal breast feeding, child growth failure, and alcohol use in early life with incidence rates, highlighting the importance of developing early-life interventions to help prevent EOCRC.

Further addressing the importance of prevention in reducing incidence and mortality of EOCRC, Lwin et al. performed a cost-effectiveness analysis of CRC screening strategies starting at 45 years of age in Germany. The authors used a simulation model to challenge the current national screening program that starts at age 50 years. The comprehensive model accounts for both adenoma and serrated pathways of CRC development, includes four screening strategies starting at age 45 (colonoscopy every 10 years, annual/biennial fecal immunochemical test, or the combination of the two), and three adherence scenarios (perfect adherence, current adherence, and high adherence which differed across testing options). Their findings demonstrate substantial gains in quality-adjusted life-years with a modest increase in costs and support initiating CRC screening in Germany at age 45 with either colonoscopy alone or combined with fecal immunochemical test. This robust simulation model can serve as an example for other countries to perform cost-effectiveness analyses to evaluate the health and economic benefits of lowering CRC screening starting age to 45 years.

Another critical point to reduce disparities along the EOCRC continuum is research on treatment and survival. Lawler et al. performed a systematic review and meta-analysis comparing tumor markers in early- vs. late-onset CRC with the understanding that clinicopathological and molecular characteristics of tumors may influence prognosis and response to treatment. Their review of 149 articles goes beyond the basic tumor markers and includes oncogene mutations, histological subtypes, microsatellite instability status, anti-tumor immunity, and the consensus molecular subtypes. This study identified that EOCRC is associated with aggressive histological subtypes (including mucinous and signet ring cell carcinomas), as well as *TP53* and *PTEN* mutations that may serve as future therapeutic targets. Continuing with the theme of EOCRC treatment, Popp et al. highlight important demographic and socioeconomic factors that are associated with time to treatment initiation for individuals with rectal cancer. Utilizing the National Cancer Database, they included >280,000 patients with rectal cancer in their overall analysis, as well as a subgroup analysis comparing early- vs. late-onset rectal cancer They found significant delays in time to treatment for rectal cancer (time to surgery, radiation, and chemotherapy) for Hispanic patients compared to non-Hispanic patients. In addition, Black patients experienced longer wait times for both radiation and chemotherapy compared to White patients. Important to our Research Topic, these disparities continued to be present in the subgroup analysis by age (<50 vs ≥50) further supporting the need to develop effective strategies to reduce treatment gaps in all patients with CRC.

The final study included in this Research Topic addresses predicting overall survival in patients with later stage EOCRC. Yin et al. developed a novel nomogram for predicting overall survival in stage III/IV EOCRC utilizing the Surveillance, Epidemiology, and End Results (SEER) database. They identified eight variables that were independent predictors of overall survival in this sample and successfully established risk stratification based on the total risk score determined by their nomogram. This information has the potential to significantly impact shared decision-making between providers and their patients with EOCRC with respect to treatment options.

In order to reduce incidence, mortality, and disparities in EOCRC, we need to continue with innovative research along the care continuum (prevention, diagnosis, treatment, and survival) such as represented by the studies in this Research Topic.
